# Development and application of an antigen capture ELISA assay for diagnosis of Japanese encephalitis virus in swine, human and mosquito

**DOI:** 10.1186/1743-422X-9-4

**Published:** 2012-01-06

**Authors:** Li Mei, Peng Wu, Jing Ye, Guangping Gao, Lin Shao, Shaomei Huang, Yaoming Li, Xiaohong Yang, Huanchun Chen, Shengbo Cao

**Affiliations:** 1 State Key Laboratory of Agricultural Microbiology, Huazhong Agricultural University, Wuhan, Hubei 430070, China; 2 Laboratory of Animal Virology, College of Veterinary Medicine, Huazhong Agricultural University, Wuhan, Hubei 430070, China; 3 Department of Clinical Laboratory, Taihe Hospital, Hubei University of Medicine, 16 Shiyan, Shiyan 442000, China; 4 Department of Animal Science and Technology, Hebei Normal University of Science & Technology, Qinhuangdao 066600, China

**Keywords:** Japanese encephalitis, Japanese encephalitis virus, Monoclonal antibody, Polyclonal antibody, Antigen capture ELISA

## Abstract

**Background:**

Japanese encephalitis (JE) is a serious zoonosis caused by the Japanese encephalitis virus (JEV) which is a mosquito-borne pathogen of the family *Flavivirus*. However, the application of several developed laboratory methods for the detection of JEV antigens or antibodies are limited by their requirements of laboratory operations, skilled technicians and special facilities.

**Results:**

To develop a method for detecting JEV antigen in swine, human, mosquito and other clinical specimens specifically, conveniently and effectively, an antigen capture enzyme-linked immunosorbent assay (ELISA) was established in this study. Sensitivity, specificity, repeatability and stability of the developed method were evaluated, and 60 clinical samples were tested in this study. The results demonstrated that the antigen capture ELISA was capable in detecting JEV antigen with high sensitivity and specificity compared with conventional methods. 14 samples showed the positive result with coincidence rate of 70%, and 46 displayed negative result with coincidence rate of 100% as compared to that of reverse transcription-polymerase chain reaction (RT-PCR).

**Conclusions:**

The developed ELISA assay provides a convenient and specific method for the large-scale determination of JEV antigen in infected swine, human and mosquito samples with high sensitivity and specificity.

## Background

Japanese encephalitis (JE) is a serious mosquito-borne zoonosis caused by the Japanese encephalitis virus (JEV) which threatens public health in southern and eastern Asia. In general, JEV is maintained in a transmission cycle between amplifier swine and vector mosquitoes [1]. As a dead-end, humans are infected by bites of infectious mosquitoes and subsequently develop neurological diseases with an estimated 10,000 JE-related deaths annually [2-5]. As an important pathogen in swine, it also induces terrible consequences in sows reproduction and death in piglets [6,7].

JEV, a member of the *Flaviviridae *family, contains a single positive 11-kb RNA genome with three structural proteins and seven nonstructural proteins [8,9], in which, E protein is the major immunogenic protein of JEV. It has the ability to induce neutralizing antibodies and is recognized as a protein candidate for the development of vaccines and diagnosis methods [10].

Several laboratory methods have been developed for the detection of JEV infection, such as virus isolation, RT-PCR. However, even with the most advanced laboratory facilities, JEV cannot be isolated from clinical specimens easily, probably because of low circulating viral numbers, soon clearance of transient viremia after onset of illness and the rapid production of neutralizing antibodies [11,12]; in addition, RT-PCR requires experienced technicians and specialized laboratory equipment, and serological test such as Hemagglutination inhibition test (HI), only could be used to detect serum antibody levels or to monitor the immunization situation. More importantly, these methods are not appropriate for investigating large numbers of samples and detecting of the level of JEV antigens in variety kinds of clinical samples.

Currently, enzyme-linked immunosorbent assay (ELISA) has been used widely for the detection of JEV infection. For example, antibody capture ELISA had been applied to detect IgM of JE in serum [13], Eiji Konishi established ELISA for quantifying antibodies against JEV nonstructural 1 protein to detect subclinical infections in vaccinated horses [1], and Sithiprasasna R developed an indirect ELISA for detecting *flavivirus *antigen in mosquitoes [14], however, the application of highly specified ELISA method for the detection of JEV antigen in swine, human, mosquito and other clinical samples uniquely and effectively hasn't been reported yet.

In this study, we aimed to develop a highly specific, sensitive, and economical antigen capture ELISA assay for detection of JEV antigen in swine, human, mosquito and other clinical samples, in an attempt to provide an effective tool for diagnosis of JEV infection.

## Results

### Production and characterization of MAb and PcAb against JEV

**Figure 1:**
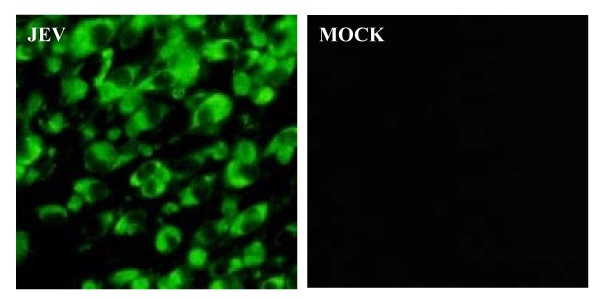
**PcAb reactionogenicity and specificity were identified by IFA**. BHK-21 cell incubated with JEV for 1 h at 37°C. At 72 h post-infection, cells were fixed with absolute methanol and treated for IFA with the prepared PcAb against JEV. Normal BHK-21 without infected JEV as a control (MOCK).

A highly specific monoclonal antibody (MAb) against JEV E protein, named 4D1, had been confirmed to take on strong immunoreactivity with E protein by Western blot and immunofluorescence assay (IFA) [10]. And we found that the reaction titer of the polyclonal antibody (PcAb) produced could also reach up to 1:20000 through indirect ELISA test. As shown in Figure 1, a strong fluorescent signal could be detected in the JEV-infected BHK-21 cells in the IFA. By checkerboard titration, optimal concentrations of the primary antibody (E MAb) and the detection antibody (PcAb) were defined as 5 ug/ml and 0.2 mg/ml, respectively.

### Sensitivity of the ELISA assay

**Figure 2:**
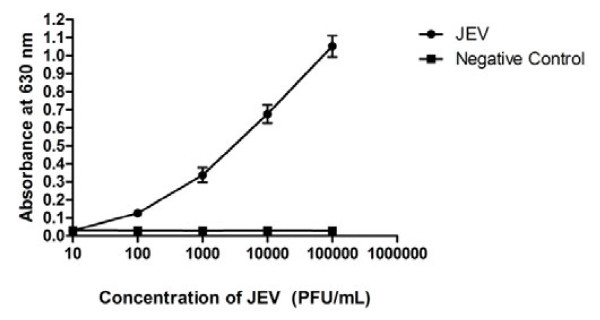
**Sensitivity of antigen capture ELISA assay**. 1 × 10 ^6 ^PFU of JEV was 10-fold diluted serially (1:10 to 1:100,000) for the test; the homogenate from blank mouse brain was used as the negative control.

1 × 10^6 ^PFU/ml JEV was serially diluted and tested by ELISA test. As shown in Figure 2, a standard curve for the 10-fold diluted 1 × 10^6 ^PFU/ml JEV test was constructed and the homogenate from blank mouse brain was used as the negative control to establish the baseline. The result showed that the minimum virus amount for detection was 1.0 × 10^4^PFU.

### Specificity of the ELISA

To evaluate the specificity of the ELISA assay, other viruses relating with popular viral diseases were subjected to this assay. As comparison, all the virus samples were also detected by RT-PCR. As shown in Table 1, positive results were observed in standard positive control and negative results were shown in PRV, PCV, PRRSV, PPV, CSFV and SIV. In addition, a weak positive signal was detected in one of the five inactivated WNV samples. The cross-reactivity between JEV and WNV was difficult to avoid because of the high homology of them. However, the specificity of the ELISA test would not be interfered by this weak signal of cross-reactivity.

**Table 1:** Specificity of ELISA assay for different virus samples compared with RT-PCR

Virus(strain)	Total	ELISA^a^	RT-PCR^b^
		
		Positive/negative	Positive/negative
JEV(P3)	15	13/2	15/0
PRV	7	0/7	0/7
PCV	8	0/8	0/8
PRRSV	8	0/8	0/8
PPV	6	0/6	0/6
WNV(inactivated)	5	1/4	0/5
CSFV	9	0/9	0/9
SIV	5	0/5	0/5
Healthy control	15	0/15	0/15

^a ^Antigen capture ELISA assay

^b ^Reverse transcription polymerase chain reaction

### Repeatability, stability and shelf time of the ELISA assay

The repeatability of the ELISA assay was evaluated with five samples. The coefficients of variation of three samples tested in this assay were 6.7%, 6.1% and 7.6% respectively (Table 2). Additionally, there was no distinct difference discovered in multiple times detection of each sample with two coated microtiter plates by significance analysis (*P *> 0.05), which confirmed the high repeatability of this ELISA assay. In order to appraise the stability of the assay, the plates coated with MAb 4D1 were stored at 37°C. The OD630 values of the standard positive control and the standard negative control were shown with no significant decrease for at least 5 days (Table 3). In addition, the plates coated with the E MAb 4D1 could be stored at 4°C or -20°C for up to 6 months without any deterioration of the coating antibody (Table 4). These data suggested that the ELISA assay has good repeatability, and the stability and shelf time could reach to the standards of ordinary ELISA tests.

**Table 2:** Repeatability of ELISA assay for 5 different samples

	Detection No. ELISA OD630									
**Specimen No**.	Plate 1			Plate 2			AVG	SD	CV	*p-Value*
					
	1	2	3	4	5	6				
1	0.46	0.499	0.535	0.475	0.442	0.49	0.484	0.0325	6.70%	0.1626
2	0.231	0.204	0.207	0.211	0.213	0.192	0.21	0.0128	6.10%	0.2346
3	0.126	0.126	0.125	0.148	0.12	0.127	0.129	0.0098	7.60%	0.2577

*AVG *Average of 6 detection OD630 values for each sample

*SD *Standard deviation of 6 detection OD630 values for each sample

*CV *Coefficient of variation of 6 detection OD630 values for each sample

*p-Value *The significance difference analysis between plate 1 and plate 2 as inter-repeat assay

**Table 3:** Stability of ELISA assay for 5 days at 37°C

	Positive Control ELISA OD630 (mean + SD)	Negative Control ELISA OD630 (mean + SD)
Day 1	0.894 ± 0.008	0.150 ± 0.012
Day 2	0.888 ± 0.004	0.145 ± 0.007
Day 3	0.852 ± 0.027	0.144 ± 0.034
Day 4	0.796 ± 0.045	0.167 ± 0.051
Day 5	0.738 ± 0.007	0.129 ± 0.002

**Table 4:** Shelf life of ELISA assay at 4°C and -20°C

	**4**°**C**		-20°C	
	
	Positive Control (mean+SD)	Negative Control (mean+SD)	Positive Control (mean+SD)	Negative Control (mean+SD)
One Month	0.962 ± 0.037	0.168 ± 0.009	0.898 ± 0.058	0.155 ± 0.061
Two Months	0.934 ± 0.041	0.155 ± 0.014	0.845 ± 0.025	0.147 ± 0.039
Three Months	0.878 ± 0.014	0.145 ± 0.023	0.797 ± 0.006	0.115 ± 0.011
Four Months	0.863 ± 0.023	0.127 ± 0.008	0.752 ± 0.009	0.104 ± 0.008
Five Months	0.856 ± 0.005	0.139 ± 0.012	0.793 ± 0.052	0.113 ± 0.043
Six Months	0.832 ± 0.034	0.143 ± 0.051	0.721 ± 0.048	0.133 ± 0.029

### Clinical sample detection

By RT-PCR analysis, 20 out of 60 clinical samples were positive and 40 were negative. Whereas the ELISA test showed 14 positive and 46 negative results with the positive coincidence rate of 70%, and the negative coincidence rate of 100% (Table 5).

**Table 5:** Results of JEV detection by ELISA assay and RT-PCR for 60 clinical samples

	RT-PCR^b^		
	
	Positive	Negative	Total
ELISA^a^			
Positive	14	0	14
Negative	6	40	46
Total	20	40	60

Compared to RT-PCR, the positive coincidence rate of ELISA:

14/(14 + 6) × 100% = 70%; the negative coincidence rate of ELISA: 40/(40 + 0) × 100% = 100%; the total coincidence rate: (14 + 40)/60 = 90%;

^a ^Antigen capture ELISA assay

^b ^Reverse transcription polymerase chain reaction

## Discussion

JE is a severe zoonosis which induces fever, aseptic meningitis, acute flaccid paralysis or classic meningomyleoencephalitis, and up to 50% of persons who survive it may have prolonged neurological or psychiatric sequelae [15-18]. Currently, there is an urgent need of a rapid diagnosis method for detection of JEV infection in both humans and animals. Since the inoculation of JEV vaccines contributes to the result of antibody-positive, the accurate diagnosis still depends on the antigen detection. However, the application of conventional methods, such as RT-PCR and viral isolation, are limited by their requirements of laboratory operations, skilled technicians and special equipments. Therefore, a more simple and rapid method for the detection of JEV antigen is needed to be developed.

Herein, we reported a convenient antigen capture ELISA for the diagnosis of JEV infection by using the MAb 4D1 against E protein of JEV and the PcAb of JEV derived from the rabbits. Because the JEV-specific primary antibody and anti-antibody are used, the proposed ELISA in this report is shown to hold the characteristic of detecting JEV antigen specifically, which effectively reduces and clears the interference from other flavivirus for JEV diagnosis. In addition, as the major immunogenic gene, E gene is highly conservative in different JEV genotypes. It has been shown that the E MAb produced by Yaoming Li according to the nucleotide sequence of E gene of JEV genotype III possessed sensitive reaction with prevalence-predominant JEV genotype I [10], which suggested that our ELISA assay could be applied to clinical detection of different JEV genotypes.

Specific binding between antibody and antigen is the most important factor for the successful development of ELISA assay. In previous studies, specificity of the test has been modified by use of monoclonal antibodies of single strains or multiple strains. However, polyclonal antibodies are typically chosen to enhance sensitivity [19,20]. Therefore, in this study, the polyclonal antibody rather than the monoclonal antibody against JEV is applied for conjugating with the antigen as detection antibody, since our results have showed that polyclonal antibodies could recognize more JEV strains, which improves the sensitivity of test.

Taken together, the ELISA assay developed in this study has following potential advantages. First, unlike RT-PCR and other viral isolation methods, our ELISA assay does not require expensive reagents and special facilities, and it can be routinely performed in any ordinary laboratory because of its simplicity and rapidity. Second, the ELISA assay has been confirmed with relatively high sensitivity. The minimum detection level of our ELISA assay (1.0 × 10^4 ^PFU) is lower than that of RT-PCR (3.2 × 10^2 ^PFU), but higher compared with that of immunochromatographic strip (ICS) (2.5 × 10^5 ^PFU) [10]. Since the ineluctability of false positive reaction by RT-PCR and the low sensitivity of ICS always prevent the applying of these methods for accurate diagnosis of JEV, our ELISA assay may be developed as an alternative method for fast diagnosis of JEV infection. Additionally, the higher sensitivity with our capture ELISA for detecting JEV antigen than with traditional ELISA has been confirmed in our assay, it could be due to introducing of a horseradish peroxidase (HRP)-labeled goat anti-rabbit antibody for specifically identifying detection antibody, which has amplified the reaction signals in whole system and modified the sensitivity. Most importantly, this ELISA assay provides a convenient way for detecting JEV in a large number of clinical specimens including swine, human and mosquito, which has not been reported before. Therefore, this method will have a broad application prospect in large-scale clinical diagnosis of JEV.

However, there are still some problems cannot be ignored. Samples collected at an appropriate time is required in this ELISA test when the virus in the detected samples has not been degraded. Because the timing of sample collection impacts on the ability to confirm the diagnosis of JEV [21] and that is necessary for successful detection by any technique. Moreover slight infection with JEV may not be capable of being detected by our ELISA test, it is due to the fact that some samples lightly infected with JEV have negative results with this ELISA but positive results by RT-PCR which has ability to detect lower virus load. Thus the ELISA test in this study is more suitable for diagnosis of severe JEV infection during an exhaustive outbreak in farm. It is necessary that these suspected samples without classical symptom of JEV infection should be further confirmed by other techniques, such as histological, immunological, or molecular assays.

## Conclusions

In summary, we successfully developed an antigen capture ELISA assay which could detect JEV antigen in swine, human, mosquito and other samples specifically and effectively, indicating that this ELISA assay could be employed as a fairly simple and efficient tool for clinical detecting and controlling JEV infection.

## Methods

### Virus preparation

JEV wide type strain P3 was propagated in brains of suckling mice and the negative control was prepared with the blank mouse brain. Pseudorabies virus (PRV, Ea strain), Porcine circovirus (PCV-2, Yu-A strain), Porcine reproductive and respiratory syndrome virus (PRRSV, YA strain), Porcine parvovirus (PPV), West Nile virus (WNV, inactivated), Classical swine fever virus (CSFV, CWH strain) and Swine influenza virus (SIV) were stored in the laboratory.

### Antibodies preparation

The MAb against JEV E protein was produced in our laboratory as reported by Li et al. [10]. The harvested MAb was purified by NAbTM Protein A/G Spin Assay (Thermo Scientific, Rockford, USA) according to the manufacturer's instructions. To produce the PcAb against JEV, rabbits were immunized subcutaneously with emulsified JEV vaccine by Freund's incomplete adjuvant (SIGMA, USA). After the fourth immunization, blood was collected and serum was separated. The PcAb was harvested and purified from the serum. Its activity was characterized by IFA.

### The assembling of the ELISA

The MAb was diluted to 5 ug/ml in coating buffer (0.05 M sodium carbonate, pH 9.5-9.7) and added into the 96 wells of polystyrene microtiter plates (KeQian Animal Biological Products Co., Ltd, Wuhan, China). After overnight incubation at 4°C, the plates were washed five times with rinse solution (0.05% Tween-20 in PBS). Nonspecific binding was blocked with blocking solution (1% bovine serum albumin in PBS) at 37°C for 1 h. Subsequently, 100 ul/well of serial diluted samples, were added in. After 30 min of incubation at 37°C, 100 ul of the PcAb against JEV, diluted in 1:10000 by blocking solution, was added as a detection antibody. After another 30 min incubation at 37°C, horseradish peroxidase (HRP)-labeled goat anti-rabbit IgG (BOSTER, Guanshan Road, Wuhan, China) was added and incubated for 30 min. Between each step, plates were washed five times in rinse solution for 2 min. After the conjugation step, tetramethylbenzidine (TMB) was added, and the result was measured by a spectrophotometer at a wavelength of 630 nm.

### The cut-off standard and detection criteria of ELISA assay

In this study, a background subtraction method was used as the border value between positive and negative, which referred to the manufacturer's instructions of PRRSV Ab ELISA Manual (Ver.1.0) PRRSV-VR/PRRSV-LA (VDPro, JENO Biotech Inc., Korea).

Test validation: OD mean of positive control (PC):0.4 or above OD mean of negative control (NC):0.2 or less

Calculation of CPC (Corrected Positive Control): CPC = OD mean of PC - OD mean of NC

Calculation of SP (Sample to Positive ratio): SP = (OD mean of sample - OD mean of NC)/CPC

The Cut-off standard of our method was set as follows:

A sample was defined as positive if the SP value was more than 0.4 and as negative if the SP value was less than 0.2. When SP value was between 0.2 and 0.4, the sample was defined to be suspect. If the detection value is suspect, the samples are needed to be collected from the herd after 1 month and tested again for confirmation.

### Sensitivity and specificity tests of the ELISA

JEV stock (1 × 10^6 ^PFU/ml) was diluted 10-fold successively. Each dilution was applied to the ELISA test. The sensitivity was determined by finding the end point dilution according to the cut-off standard prescribed. The specificity of the ELISA was evaluated by detecting several different viruses including PRV, PCV, PRRSV, PPV, inactivated WNV, CSFV and SIV. 1.0 × 10^6 ^PFU/ml JEV strain was used as positive control, while the homogenate from healthy mouse brain was used as negative control.

### Repeatability, stability and shelf time evaluation of the ELISA assay

In regard to the repeatability evaluation, the ELISA assay was utilized to detect 3 samples with two groups of coated microtiter plates. Each sample was tested three times separately in one plate for intra-repeat assay and results in two plates were regarded as inter-repeat. The differences were evaluated through comparing variation coefficient and analyzing statistical significance. To analyze the stability of the ELISA assay, the coated polystyrene ELISA plates were placed in 37°C incubator. Standard positive control and negative control were detected by one ELISA plate each day. To determine the shelf life of the ELISA assay, the coated ELISA plates were put into 4°C and -20°C respectively for several months. One piece of the coated plate was used to detect the standard positive control and negative control each month.

### Detection of clinical samples in field

60 clinical samples (including 20 mosquito homogenate, 24 swine brain tissues and 16 cerebrospinal fluid of the human patients) were detected by ELISA assay. Among which, 16 cerebrospinal fluid specimens from the human patients at Taihe Hospital (Shiyan, China) were collected on admission; 24 swine brain tissues were collected from clinical detection; 20 mosquito species were caught by the mosquito catcher. The clinical samples from the mosquito, swine, and cerebrospinal fluid were all stored at -80°C before assay. Five grams of brain tissue of swine, mosquitoes in 5 ml of medium were homogenized in a sterile grinder and centrifuged at 10,000 × g for 10 min respectively. The supernatant was filtered with 0.22 um filter membrane and the percolates collected as tissue samples. RT-PCR was applied as the comparative methods.

### Statistical analysis

Statistical analysis was subjected to a Student's *t *test. *P *> 0.05 was considered to be no significance of difference.

## Abbreviations

JE: Japanese encephalitis; JEV: Japanese encephalitis virus; ELISA: Enzyme-linked immunosorbent assay; HI: Hemagglutination inhibition test; MAb: Monoclonal antibody; PcAb: Polyclonal antibody; RT-PCR: Reverse Transcription-polymerase chain reaction; ICS: Immunochromatographic strip; HRP: Horseradish peroxidase; TMB: Tetramethylbenzidine; PC: Positive control; NC: Negative control; CPC: Corrected positive control; SP: Sample to positive ratio.

## Competing interests

The authors declare that they have no competing interests.

## Authors' contributions

LM and PW carried out most of the experiments and wrote the manuscript. JY, GG, LS, SH, YL and XY participated part of experiments and samples collection. HC and SC conceived of the study, participated in its design and coordination, and revised the manuscript. All authors read and approved the final manuscript.
